# Nutrigenomic insights and cardiovascular benefits of blackberry (*Rubus ulmifolius* Schott.) and mugwort (*Artemisia campestris* L.)

**DOI:** 10.1113/EP092218

**Published:** 2025-04-24

**Authors:** Afaf Mehiou, Anca Lucau‐Danila, Zachee L. E. Akissi, Chaimae Alla, Nourelhouda Bouanani, Abdelkhaleq Legssyer, Jean‐Louis Hilbert, Sevser Sahpaz, Abderrahim Ziyyat

**Affiliations:** ^1^ Laboratory of Bioresources, Biotechnologies, Ethnopharmacology and Health, Department of Biology, Faculty of Sciences University Mohammed First Oujda Morocco; ^2^ BioEcoAgro Joint Cross‐Border Research Unit, UMRt 1158 University of Lille Lille France; ^3^ Joint Laboratory CHIC41H, University of Lille‐Florimond Desprez Villeneuve d'Ascq France

**Keywords:** *Artemisia campestris*, bioactive compounds, gene expression, hypotension, *Rubus ulmifolius*, vasodilatation

## Abstract

Blackberry (*Rubus ulmifolius* Schott) and mugwort (*Artemisia campestris* L.) are plants traditionally used to treat various pathologies, including hypertension. The vasodilatory and hypotensive effects of blackberry were investigated through experiments in rat models (*n* = 5 rats per group) and compared with those of mugwort, which had been demonstrated previously. A nutrigenomic experiment in mouse models (*n* = 3 mice per group) was also performed for both plants to associate biomarker genes with these effects. Additionally, a phytochemical analysis was carried out to identify the bioactive molecules responsible for the cardiovascular effects. A dose‐dependent hypotensive effect and a carbachol‐like vasodilatory effect were observed for blackberry and compared with those of mugwort. These effects were associated with the deregulation of gene expression related to vessel lumen expansion (*Amotl2*, *Cdh1* and *Tfcp2l1*) and circulatory system morphology and activity (*Dsp*, *Ahnak*, *Prcp* and *Smtnl2*) for both plants. Their functional potential also includes antiproliferative, antimicrobial, anti‐inflammatory and appetite‐regulating properties. Chlorogenic acids, quercetin and kaempferol derivatives were identified in blackberry as the main bioactive molecules likely to be responsible for its cardiovascular effect. The blackberry extract exhibited a vasorelaxant effect 20 times greater than mugwort, attributed to the exclusive presence of the hypotensive galloyl‐*bis*‐HHDP glucose derivative and a more pronounced upregulation of *Tfcp2l1*, which is involved in epithelial cell maturation. This study validates the traditional use of blackberry and mugwort in treatment of hypertension, identifies marker genes and bioactive molecules for vasodilatory and hypotensive effects and expands their potential applications to cancer prevention, inflammation reduction and appetite regulation.

## Abstract (highlights)

- **What is the central question of this study?**
  Are the hypotensive and vasodilatory effects of blackberry comparable to those of mugwort, and which biomarker genes are associated with these effects?
- **What is the main finding and its importance?**
  We found evidence of significant hypotensive and vasodilatory effects of blackberry, which were more pronounced than those of mugwort. The exclusive presence of the galloyl‐*bis*‐HHDP glucose derivative in blackberry, known for its hypotensive properties, along with more pronounced upregulation of *Tfcp2l1* (implicated in epithelial cell maturation) in mice after ingestion could explain the enhanced cardiovascular effects of blackberry compared with mugwort.

## 1 INTRODUCTION

Cardiovascular diseases are the leading cause of morbidity and mortality worldwide. Hypertension is a prominent risk factor in the development of cardiovascular diseases, characterized clinically by a systolic blood pressure (SBP)/diastolic blood pressure (DBP)  ≥ 140/90 mmHg (Brouwers et al., 2021; Giles, 2006). Hypertension is categorized into two types: essential, also known as primary; and secondary. Essential hypertension is the most common form, accounting for 90% of all hypertension cases, and its exact causes are not identified. Secondary hypertension is rare, being identified in 10% of patients with high blood pressure, and can be attributed to kidney diseases (renovascular and renal parenchymal diseases), adrenal conditions (such as Cushing's syndrome and pheochromocytoma), medications or alcohol (Carey et al., 2018). Hypertension increases the risk of developing cardiovascular, cerebrovascular or neurodegenerative complications, in addition to renal dysfunction. The risk of cardiovascular mortality doubles for each increase in SBP/DBP of 20/10 mmHg (Carey et al., 2018; Crim et al., 2012). The global prevalence of hypertension was ∼25% (972 million individuals) in 2000 (Kearney et al., 2005). This number increased to 1.28 billion by 2019, and it is projected to reach ∼1.56 billion by 2025 (Kearney et al., 2005).

To reduce cardiovascular risks associated with elevated blood pressure, both non‐pharmacological and pharmacological treatments are necessary. Non‐pharmacological therapy relies on lifestyle and dietary measures, including weight reduction, sodium restriction, alcohol restriction and physical exercise. Pharmacological therapy includes angiotensin II receptor antagonists, angiotensin‐converting enzyme inhibitors, diuretics, calcium channel blockers and vasodilators, which serve as the main antihypertensive agents (Brouwers et al., 2021; Eschenhagen, 2017). Additionally, in various regions worldwide, including Morocco, herbal medicine is used, using medicinal plants to address various health concerns, such as hypertension (Ziyyat et al., 1997).

In Morocco, medicinal plants have been used since ancient times to address various ailments (Elachouri, 2018). According to ethnobotanical surveys conducted in various regions, Moroccans use a wide variety of plant species for treating high blood pressure (Eddouks et al., 2002; Idm'hand et al., 2019; Insaf et al., 2023; Jouad et al., 2001; Lyoussi et al., 2023; Tahraoui et al., 2007; Ziyyat et al., 1997). Among these plants, *Allium sativum* L., *Arbutus unedo* L., *Urtica dioica* L., *Olea europea* L. and *Artemisia campestris* L. have been identified (Ziyyat et al., 1997). Mugwort (*Artemisia campestris* L., Asteraceae) is a herbaceous and medicinal perennial plant from eastern Morocco (Dib, Angenot et al., 2017). Mugwort has been the subject of numerous phytochemical and pharmacological studies (Dib, Angenot et al., 2017; Dib & El Alaoui‐Faris, 2019). Previous research by our team demonstrated the antihypertensive and vasorelaxant effects of the aqueous extract derived from the aerial parts of mugwort, in addition to the presence of phenolic compounds, such as chlorogenic acid, 3,5‐dicaffeoylquinic acid, 4,5‐dicaffeoylquinic acid, 3,4‐dicaféoylquinic acid and vicenin 2 (Dib, Tits et al., 2017).

Blackberry (*Rubus ulmifolius* Schott.) is a thorny shrub ∼1.5 m tall, belonging to the Rosaceae family and commonly known as wild blackberry. The plant is present in Asia, Europe and North Africa, where it is used for medicinal purposes and for its edible fruits (Chauhan & Chauhan, 2022; Tofan‐Dorofeev, 2015). Traditionally, blackberry is used to treat high blood pressure (Camejo‐Rodrigues et al., 2003), mouth ulcers and diarrhoea (Bulut & Tuzlacı, 2015). In Morocco, the underground parts of the blackberry are prepared as a decoction and used to address digestive and cardiovascular issues (Fakchich & Elachouri, 2021). Beyond its traditional uses, various therapeutic properties have been attributed to blackberry, including antiproliferative (Triggiani et al., 2012), wound healing (Manca et al., 2015) and antimicrobial effects (Panizzi et al., 2002). An antihypertensive effect was also observed by Feresin et al. (2019), who demonstrated that dietary supplementation with freeze‐dried blackberries attenuated the increase SBP induced by chronic angiotensin II injection. Previous phytochemical studies on different parts of the blackberry plant have reported the presence of secondary metabolites, including flavonoids, phenolic acids, tannins and anthrones (Flamini et al., 2002; Martins et al., 2014). The limited chemical studies focused on the leaves have identified the presence of phenolic acids and flavonoids (Dall'Acqua et al., 2008; Panizzi et al., 2002). A recent literature review on this plant provides a comprehensive overview of its diverse traditional uses, phytochemistry and pharmacological effects (Mehiou et al., 2024).

Our study aimed to evaluate the full spectrum of nutrigenomic responses generated by administering aqueous extracts of blackberry and mugwort in mice. We then focused on the hypotensive and vasodilatory effects of these two plants. Previous studies conducted on a rat model reported that mugwort extract triggers a significant vasorelaxant effect, emphasizing the vascular mechanism of action (Dib, Tits et al., 2017). We aimed to determine whether the aqueous extract of blackberry leaves (AERu) is capable of producing similar effects by reproducing the same experimental conditions. For this purpose, we conducted two types of experiments on rats using AERu. First, to study the vasodilatory effect, isolated rat aorta rings were used *ex vivo* and placed in isolated organ tanks. Second, to study the hypotensive effect, experiments were conducted in vivo on anaesthetized normotensive rats by inserting a catheter into the femoral artery to measure blood pressure and another into the femoral vein for intravenous injections of either the AERu or the reference pharmacological substances. The phytochemical composition of AERu was also analysed. Cardiovascular effects of blackberry were demonstrated, discussed and compared with those of mugwort. These effects were related to gene expression and the phytochemical composition of the plants.

## 2 MATERIALS AND METHODS

### 2.1 Ethical approval

All procedures on mice were performed in accordance with Directive 2010/63/EEC for the protection of animals used in scientific research, in addition to Laws 2012‐10 (2012) and 2013‐118 (2013). These procedures were approved by the ethics committee in charge of animal experiments (CEEA 75, protocol APAFIS 25250‐2019061315316749) and certified under B5900912.

All procedures involving rats were carried out in an ethically proper way, complying with the internationally accepted *Guide for the Care and Use of Laboratory Animals*, published by the US National Research Council (National Research Council, 2011). All experimental protocols were ethically approved by the Faculty of Sciences (Mohamed First University, Oujda, Morocco; ethic approval number 7/24‐LBBES).

### 2.2 Plant material and preparation of aqueous extracts

The aerial parts of mugwort and blackberry were collected in September 2022 from Tendrara (32°49′48″N, 1°39′36″W) and Berkane (34°53′25.0″N, 2°20′44.8″W), respectively, in the Oriental region of Morocco. A voucher specimen was deposited in the Herbarium of Faculty of Sciences, University Mohamed First (Oujda, Morocco) under the reference numbers HUMPOM‐151 and HUMPOM741, respectively. The name of both plants has been verified at http://www.theplantlist.org as of 30 June 2024.

The collected parts were dried, then ground into powder. Twenty grams of powder was added to 100 mL of boiling distilled water. The obtained extract (infusion) was carefully filtered and subsequently evaporated using a rotary evaporator at 50°C until a reduced volume of concentrated extract was achieved. This concentrate was then distributed into a Petri dish and dried in an oven at 40°C. After drying, the extract was stored in a freezer at −20°C until use. The extract yield was calculated using the following formula: yield % = (M1/M0) × 100, where M0 is the mass of the dried plant, and M1 is the mass of the extract after evaporation.

### 2.3 Phytochemical screening of AERu

The identification of phenolic acids, flavonoids and tannins in the AERu was carried out using the thin layer chromatography method. Phytochemical screening used silica plates (Macherey Nagel DC‐Fertigfolien ALUGRAM Xtra SIL G/UV_254_) as the stationary phase and a mobile phase consisting of ethyl acetate/acetic acid/formic acid/water (100:11:11:20). Specific reagents, including Naturstoff reagent (Neu) for flavonoids and FeCl_3_ at 1.3% (v/v) for tannins, were used to identify specialized metabolites. Ten milligrams of AERu was dissolved in 1 mL of methanol, and 10 µL of this solution was applied to the plate deposition line using a capillary. After drying, the plate was placed in a tank containing the mobile phase, allowing the migration of components based on their affinity between the two phases. Silica plates were visualized under visible light and ultraviolet light at wavelengths of 254 and 354 nm, both before and after detection.

### 2.4 Quantification of total polyphenols and total tannins of the AERu

For the quantification of polyphenols, 0.63 mg of AERu was dissolved in 1.5 mL of water and heated in a water bath at 95°C for 30 min. After heating, the mixture was centrifuged at 5000 rpm for 5 min. A 500 µL aliquot of the supernatant was taken for the determination of total polyphenols. Another 500 µL aliquot of the supernatant was mixed with 10 mg of slightly chromated hide powder (Merck). This mixture was shaken for 1 h in the dark, then centrifuged again at 5000 rpm for 5 min. The resulting supernatant was used to measure the total non‐tannic phenolic compounds (polyphenols not adsorbed by the slightly chromated hide powder). The total tannin content was calculated by subtracting the amount of total non‐tannic phenolic compounds from the total polyphenols.

The quantification of total polyphenols and total tannins was conducted using microplate spectrophotometry, based on the colorimetric Folin–Ciocalteu method, with some modifications. A standard curve was prepared using gallic acid in distilled water at concentrations of (0, 6.25, 12.5, 25, 50 and 100 mg/L). A mixture of 20 µL of extract (0.42 mg/mL in distilled water) or gallic acid and 100 µL of Folin reagent (diluted 1:10) was prepared. After mixing well for 4 min, 80 µL of sodium carbonate (Na_2_CO_3_) at 106 g/L prepared in distilled water was added. Absorbance was measured at 760 nm against a blank of distilled water every 15 min over a period of 90 min.

All preparations were carried out in triplicate. The contents of total polyphenols and total tannins were expressed in milligrams of gallic acid equivalents per gram of extract (mg GAE/g of extract).

### 2.5 Ultra high‐performance liquid chromatography and electrospray ionization–mass spectrometry analysis

The chemical composition of AERu was analysed using an Acquity ultra high‐performance liquid chromatography H‐Class Waters System (Guyancourt, France), which included two independent pumps, a controller, a diode strip detector and a QDa electrospray quadrupole mass spectrometer. Ionization was performed in negative mode with a mass range of 50–1250 Da. The cone voltage was set at 15 V and the capillary voltage at 0.8 kV. The flow rate and column temperature were set at 0.3 mL/min and 30°C, respectively. The wavelength range was 190–400 nm, with a resolution of 1.2 nm. The stationary phase was a 2.6 µm Uptisphere C18‐AQ 2.1 mm × 100 mm column. The mobile phase consisted of the mixture of: (A) ultrapure water + 0.1% formic acid (Carlo Erba Reagents, Val de Reuil, France); and (B) acetonitrile (Carlo Erba Reagents, Val de Reuil, France) + 0.1% formic acid. One milligram of extract was solubilized in 1 mL of HPLC quality methanol, then centrifuged at 15000 rpm for 15 min. Two microlitres of the supernatant was injected. The elution gradient was as follows: from 5% to 80% (B) (0 to 9 min); 100% (B) (9.5 to 10.5 min); and 5% (B) (11 to 14 min).

### 2.6 Animal experiments

Nutrigenomic studies of male BALB/cOlaHsd 8‐week‐old mice were conducted on the PHExMAR platform of Lille University (France). The mice were randomly divided into three groups (*n* = 6 mice per group) and housed in a controlled environment (with a temperature of 22°C, a 12 h–12 h light–dark cycle and ad libitum access to standardized food and water). Separate groups of mice were fed with an aqueous extract of mugwort (AEAc) or blackberry (AERu), each at a concentration of 6 mg/mL. This solution corresponded to 150 mg of plant extract per kilogram of body weight per day, which was deemed appropriate as a moderate dietary dose. The administered dose was calculated to align with human equivalent body weight, following general guidelines derived from traditional practices and pharmacological studies (WHO, 1999–2007; Blumenthal & Busse, 1999). This approach was also consistent with previous nutrigenomic studies that used similar dosing strategies (Fouré et al., 2018). The mouse gavages consisted of a daily force‐feeding of 500 µL of AEAc or AERu solution besides the standard chow (Diet A04C‐10; Scientific Animal Food and Engineering, Augy, France) and water provided ad libitum. Control animals underwent an equivalent force‐feeding with water (Ctr). The three groups of mice (Ctr, AEAc and AERu), were nourished for 20 days. At the end of this period, three mice for each condition were euthanized by isoflurane overdose, following ethical guidelines for animal care, and the central core of the liver left lobe was cut into cubes, immediately frozen in liquid nitrogen and stored as individual samples at −80°C for transcriptomics.

For cardiovascular studies, the experiments were conducted on male, 12‐ to 14‐week‐old Wistar rats raised in standard breeding conditions in the animal house of the Department of Biology at the Faculty of Sciences in Oujda (Morocco). The rats experienced a 12 h–12 h light–dark cycle with temperature maintained at 22°C ± 2°C. They were provided with ad libitum access to standardized food (LabDiet 5001; Provimac, ONSSA Agreement no. ALC.14.4.16; Approval no. RVL 105 CL,Meknès‐Morocco) and water.

BALB/cOlaHsd8 mice were selected for the nutrigenomics experiments owing to their widespread use in genomic research, the availability of well‐characterized genetic data, and their sensitivity to dietary and metabolic interventions. Wistar rats were used for cardiovascular experiments because their larger size and well‐developed arterial system provided practical advantages and ensured comparability with previous research.

### 2.7 RNA extraction and microarray analysis

Total RNA was extracted from hepatic tissues using an RNAspin column (Macherey‐Nagel, Düren, Germany). The Agilent Whole Mouse Genome Microarray Sure Print GE 4 × 44 v.2, containing 45 220 oligonucleotide probes, was used to examine gene expression profiles. Procedures for RNA amplification, staining, hybridization and washing followed the manufacturer's guidelines. The GenePix 4000B scanner (Molecular Devices Corporation, Sunnyvale, CA, USA) was used to scan slides at a resolution of 5 µm per pixel. GenePix Pro 6.0 software was used for grid alignment and digitization of expression data. The Quantile algorithm was applied to normalize expression data. To identify genes displaying changes in expression across repetitions, a script applying library functions in R was used, and genes with a false discovery rate of <5% were retained. Fold change (FC) values were determined by comparing individual treated samples with the mean of the controls. Genes were considered differentially expressed if the fold change was >1.5 or <0.5. Functional annotations for differentially expressed genes (DEGs) were sourced from NCBI GenBank, with physiological processes assigned through NCBI, AmiGO 2 Gene Ontology and UniProt. KEGG pathway analysis was performed to identify relevant biological pathways for the selected genes. Microarray data have been submitted to the NCBI GEO: archive for functional genomics data, with the accession number GSE251733.

### 2.8 Blood pressure measurements in normotensive anaesthetized rats

The group of rats designated for in vivo experiments (*n* = 5 rats per group) were weighed (220–360 g), anaesthetized via an intraperitoneal injection of sodium pentobarbitone (50 mg/kg body mass), and placed on a heating plate to maintain a temperature of 37°C. Cannulation of the femoral vein and artery was performed. Heparinized catheters were inserted to prevent coagulation; the catheter in the inferior vena cava via the femoral vein facilitated drug administration, and the one in the abdominal aorta via the femoral artery enabled arterial signal recording and visualization using a ‘National Instrument’ data acquisition card and Labview 6.1 software. A 30 min stabilization period followed signal acquisition to stabilize parameters such as SBP, DBP, mean arterial pressure (MAP) and heart rate. The functionality of the measurement system was then confirmed by increasing blood pressure with phenylephrine (PHE; 3 µg/kg body mass) and lowering it with sodium nitroprusside (SNP; 10 µg/kg body mass). Finally, the values of SBP, DBP, MAP and heart rate were recorded before (control) and after the administration of cumulative doses of AERu (1, 5, 10, 20, 30 and 40 mg/kg). The time interval between injections was 10 min.

### 2.9 Vascular effects in rats

The group of rats designated for *ex vivo* experiments (*n* = 5 rats per group) were weighed (200–300 g), anaesthetized via an intraperitoneal injection of sodium pentobarbitone (50 mg/kg body mass), and secured on a dissection plate. An incision was made in the abdominal wall to locate the thoracic aorta, which exited the left ventricle and ran along the spine. The thoracic aorta was quickly removed and placed on paraffin in a Petri dish containing a well‐oxygenated physiological solution (95% O_2_–5% CO_2_). This solution comprised glucose (11 mM), CaCl_2_ (2.6 mM), NaCl (119 mM), MgSO_4_ (1.2 mM), NaHCO_3_ (25 mM), KCl (4.7 mM) and KH_2_PO_4_ (1.2 mM). Under a binocular microscope, the aorta was carefully stripped of adipose tissues and cut into rings measuring 3–4 mm in length. Each aortic ring was suspended between two hooks and placed in an isolated organ bath with 11 mL of oxygenated physiological solution, kept at 37°C and pH 7.4. The upper hook was connected to a force transducer, and a tension of 1 g was applied by elevating the rack. The aortic ring was then allowed to stabilize for 30 min, during which it relaxed spontaneously until reaching baseline tension. After the equilibration period, the endothelial integrity of the aorta was tested by inducing contraction with PHE (10^−6^ M) and relaxation with carbachol (CCH; 10^−4^ M). Once endothelial integrity was confirmed, the aorta was rinsed twice with physiological solution and exposed to cumulative doses (10^−3^, 10^−2^ and 10^−1^ g/L) of AERu.

### 2.10 Statistics

Nutrigenomic experiments were conducted on *n* = 3 mice per group. *Ex vivo* experiments and in vivo experiments on anaesthetized normotensive rats were performed on *n* = 5 rats per group. These sample sizes were chosen to ensure sufficient statistical power to detect meaningful differences between conditions while accounting for biological variability, in line with similar previous studies with which comparisons were made. At the same time, we balanced the need to minimize the number of animals used while adhering to ethical considerations.

The Shapiro–Wilk test was used to evaluate data normality, and Bartlett's test was used to examine homogeneity of variances. Given that the data met normality and homogeneity of variances, statistical differences were determined using one‐way and two‐way ANOVA, followed by Bonferroni's *post hoc* test. A value of *p* < 0.05 was considered significant. Data were presented as the mean ± SD using GraphPad Prism 7 software for Windows (San Diego, CA, USA).

## 3 RESULTS

### 3.1 Phytochemical characterization of AERu

**FIGURE 1:**
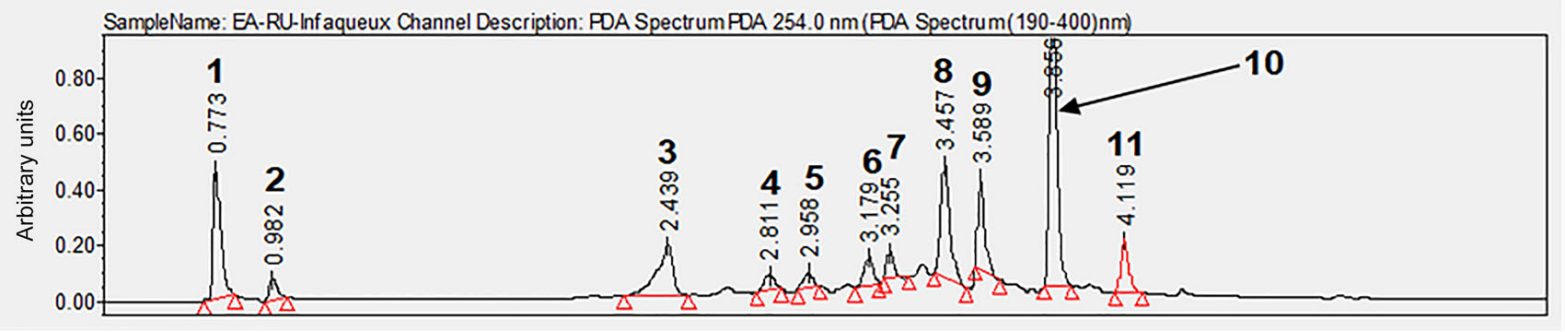
Ultra high‐performance liquid chromatography chromatogram (monitored at 254 nm) of aqueous extract of blackberry leaves. (1) Rubanthrone A; (2) alcool‐3,4‐dihydroxybenzylic and methylgallate; (3) neochlorogenic acid; (4) 3‐*p*‐coumaroylquinic acid derivative; (5) chlorogenic acid; (6) afzelin; (7) 3‐*p*‐coumaroylquinic acid; (8) galloyl‐*bis*‐HHDP glucose derivative; (9) not identified; (10) quercetin‐3‐*O*‐β‐d‐glucuronide; and (11) kaempferol‐3‐*O*‐β‐d‐glucuronide.

Phytochemical analysis of AERu confirmed the presence of phenolic acids, flavonoids and hydrolysable tannins. The results indicated that AERu was richer in tannins compared with other phenolic compounds (Table 1). Ultra high‐performance liquid chromatography analysis of AERu, recorded at 254 nm, enabled the identification of its constituent molecules (Figure 1). The identification and assignment of peaks were based on a comparison of their retention times and mass spectral data with values reported in the literature (Table 2).

**TABLE 1:** Total phenolic and tannin content of aqueous extract of blackberry leaves.

Total phenolic content (mg GAE/g)	Total non‐tannic phenolic content (mg GAE/g)	Total tannin content (mg GAE/g)
258 ± 6	91 ± 4	167 ± 2

*Note*: Values are expressed as the mean ± SD (*n* = 3 measures). Abbreviation: GAE, gallic acid equivalent.

**TABLE 2:** Ultra high‐performance liquid chromatography and electrospray ionization–mass spectrometry negative ion analysis of aqueous extract of blackberry leaves.

Peak	Retention time (min)	Absorbance (nm)	Molecular ion [M‐H]^−^ (*m*/*z*)	Tentative identification	References
1	0.735	220	215.20 and 377.17	n.i. and rubanthrone A	Flamini et al. (2002)
2	0.994	233 and 314	139.05 and 183.08	Alcohol‐3,4‐dihydroxybenzylic and methylgallate	Niero and Filho (2008)
3	2.450	325	353.16	3‐Caffeoylquinic acid (neochlorogenic acid)	Dall'Acqua et al. (2008); Panizzi et al. (2002)
4	2.826	309	337.18	3‐*p*‐Coumaroylquinic acid derivative	Karaklajic‐Stajic et al. (2023); Martins et al. (2014)
5	2.966	326	353.18	5‐Caffeoylquinic acid (chlorogenic acid)	Dall'Acqua et al. (2008); Panizzi et al. (2002)
6	3.173	361	431.40	Kaempferol‐3‐*O*‐α‐l‐rhamnopyranoside (afzelin)	Wang and Jia (1998)
7	3.325	361	337.21	3‐*p*‐coumaroylquinic acid	Karaklajic‐Stajic et al. (2023); Martins et al. (2014)
8	3.473	222	934.55	Galloyl‐*bis*‐HHDP glucose derivative	Martins et al. (2014); Mertz et al. (2007)
9	3.867	357	477.21	Quercetin‐3‐*O*‐β‐d‐glucuronide	Dall'Acqua et al. (2008); Mertz et al. (2007); Panizzi et al. (2002)
10	4.130	340	461.24	Kaempferol‐3‐*O*‐β‐d‐glucuronide	Dall'Acqua et al. (2008); Mertz et al. (2007); Panizzi et al. (2002)
11	4.346	n.i.	547.34	Kaempferol derivative	Mertz et al. (2007)
12	4.450	n.i.	711.64	Kaempferol derivative	Mertz et al. (2007)
13	4.899	n.i.	725.58	Kaempferol derivative	Mertz et al. (2007)
14	5.336	n.i.	695.64	Kaempferol derivative	Mertz et al. (2007)

Abbreviation: n.i., not identified.

### 3.2 Nutrigenomic analyses

**FIGURE 2:**
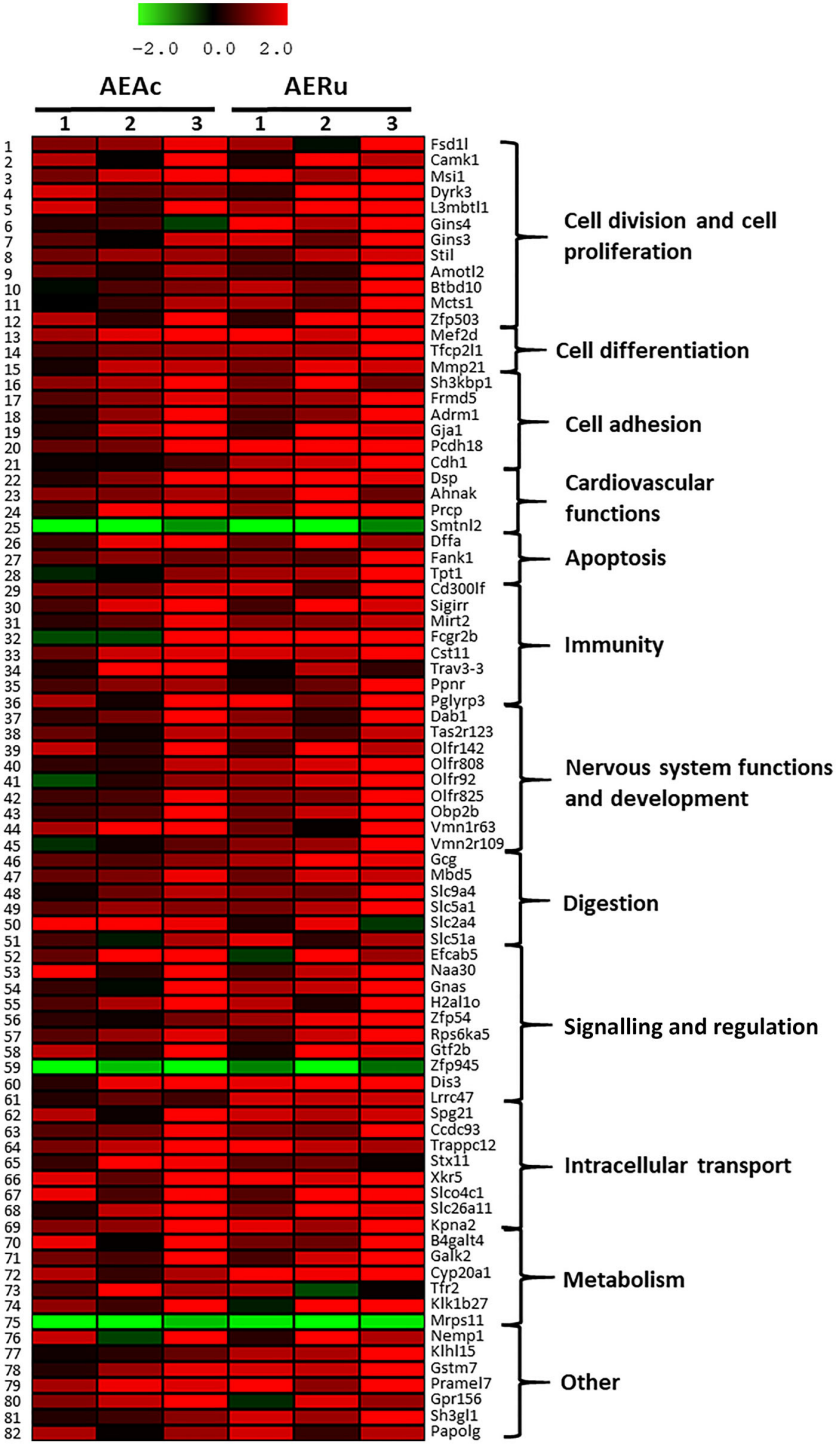
Gene expression profiles in hepatic tissue of mice after different diets. A total of 82 differentially expressed genes were retained and represented as log_2_ fold change values. The physiological processes in which they are involved are shown on the right part. Three animals per condition were analysed (1–3) for mugwort (AEAc) and blackberry (AERu) treatment.

**FIGURE 3:**
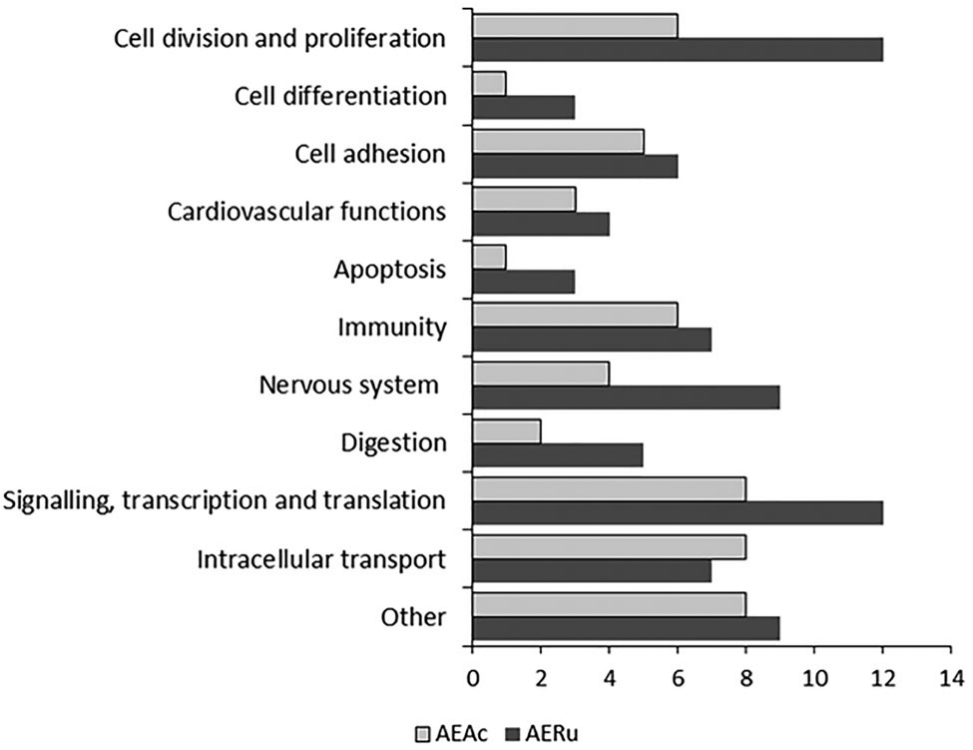
Functional classification of differentially expressed genes in hepatic tissue of mice after different diets. Genes were considered relevant for functional designation if their expression levels were deregulated similarly in at least two out of three values per condition. For 95% of genes, the three individuals analysed showed a similar response for one or both conditions. Abbreviations: AEAc, mugwort treatment; AERu, blackberry treatment.

Comparative whole transcriptomics of the liver tissue from mice force‐fed with mugwort (AEAc) or blackberry (AERu) solution allowed us to identify 82 DEGs that showed differences in transcript accumulation (Figure 2; Supplementary file). Blackberry treatment triggered a gene expression profile fairly similar to that of mugwort. Of the total 82 deregulated genes, only three genes showed downregulation in comparison to water‐fed mice (Ctr), whereas the other DEGs showed upregulation, with several variations in expression rate for the two tested plants. The retained DEGs exhibited a moderate FC, ranging from 6.88 to 0.24, probably owing to the moderate dose of the product ingested by the mice. According to Gene Ontology (GO), NCBI and KEGG annotation, these DEGs were classified into 11 functional groups (Figure 3), and the numerical variations of the DEGs in each functional group generally indicated a more pronounced response to the blackberry compared with the mugwort for most of the retained functions, especially for cell division and proliferation, immunity and nervous system functions and development.

### 3.3 Hypotensive effect of the AERu

**FIGURE 4:**
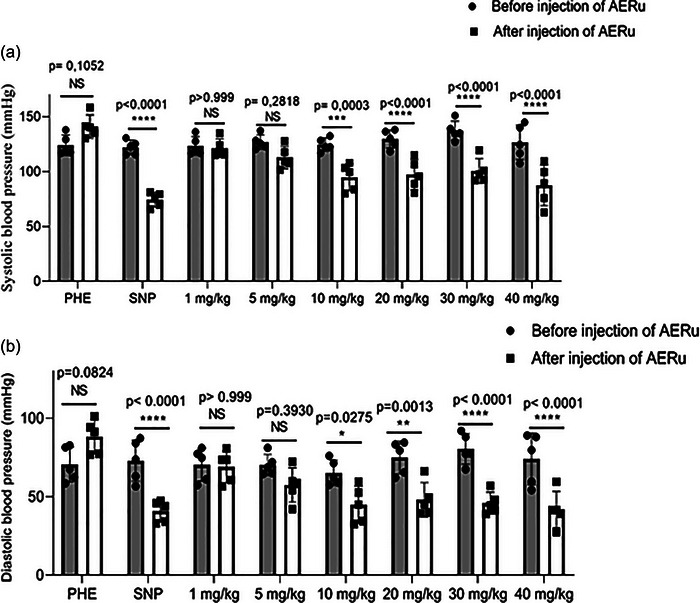
Effect of intravenous injection of an aqueous extract of blackberry leaves (AERu) on systolic blood pressure (a) and diastolic blood pressure (b) in normotensive anaesthetized rats. Data are presented as the mean ± SD (*n* = 5 rats per group). ^*^ *p *< 0.05, ^**^ *p* < 0.01, ^***^ *p* < 0.001 and ^****^ *p *< 0.0001 versus before injection of AERu. Abbreviations: NS, not significant; PHE, phenylephrine; SNP, sodium nitroprusside.

**FIGURE 5:**
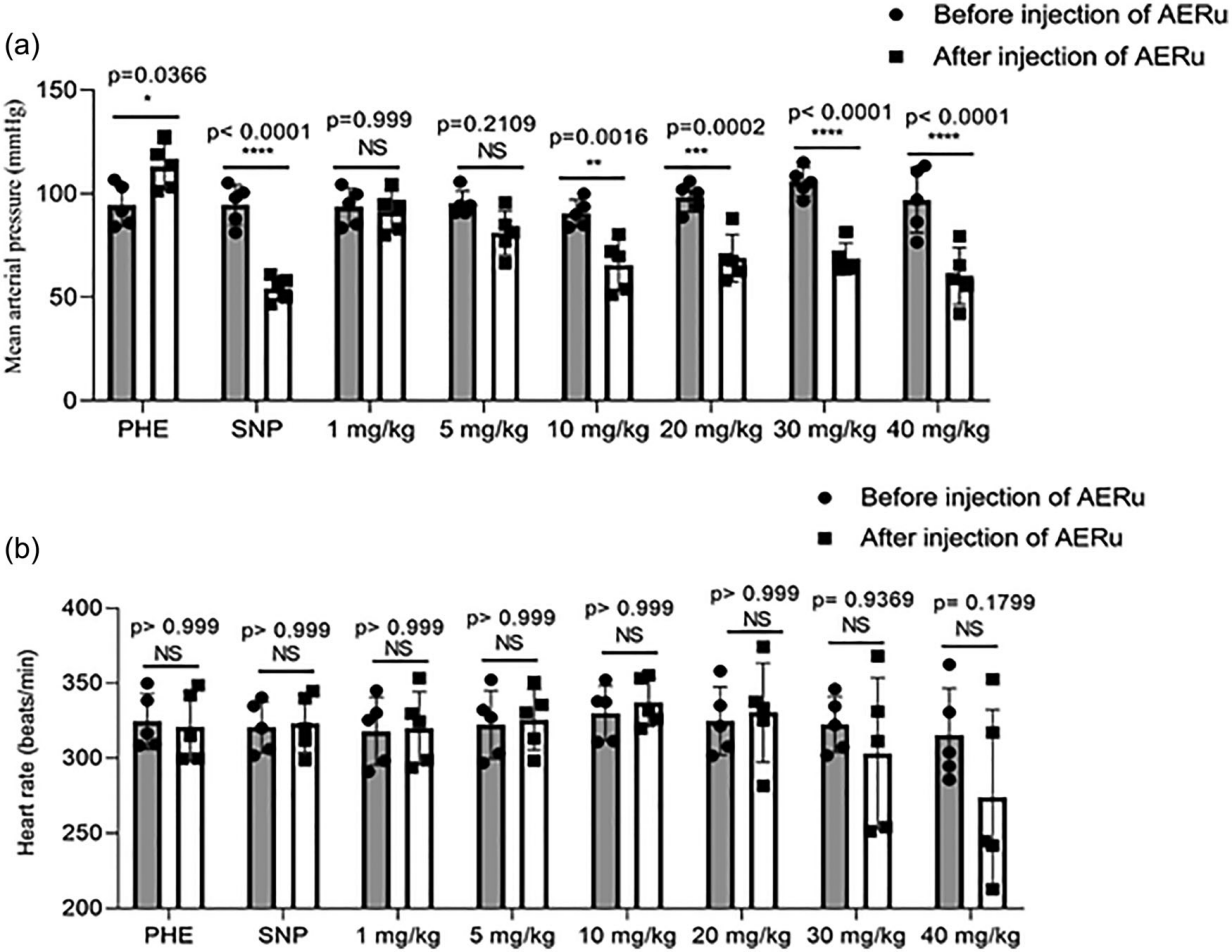
Effect of intravenous injection of aqueous extract of blackberry leaves (AERu) on mean arterial pressure (a) and heart rate (b) in normotensive anaesthetized rats. Data are presented as the mean ± SD (*n* = 5 rats per group). ^*^ *p *< 0.05, ^**^ *p* < 0.01, ^***^ *p* < 0.001 and ^****^ *p *< 0.0001 versus before injection of AERu. Abbreviations: NS, not significant; PHE, phenylephrine; SNP, sodium nitroprusside.

The intravenous injection of PHE resulted in an increase in SBP, DBP and MAP by 14%, 25% and 20%, respectively. These values returned to baseline levels, indicating normal baroreflex function. After a 10 min interval, the administration of SNP induced a decrease in these parameters by 39%, 44% and 43%, respectively, which also returned to baseline levels. These results confirmed the integrity of the baroreflex arc, a prerequisite for testing the effects of the extract (Figures 4 and 5).

The intravenous injection of AERu at doses of 1, 5, 10, 20, 30 and 40 mg/kg body mass induced reductions in SBP by 2% (*p* > 0.999, Hedges’ *g* = 0.23), 11% (*p* = 0.2818, *g* = 1.48), 24% (*p* = 0.0003, *g* = 2.74), 25% (*p* < 0.0001, *g* = 2.57), 29% (*p* < 0.0001, *g* = 3.28) and 31% (*p* < 0.0001, *g* = 2.05), respectively. Likewise, DBP decreased by 2% (*p* > 0.999, *g* = 0.12), 18% (*p* = 0.3930, *g* = 1.28), 31% (*p* = 0.0275, *g* = 1.83), 36% (*p* = 0.0013, *g* = 2.26), 43% (*p* < 0.0001, *g* = 3.65) and 44% (*p* < 0.0001, *g* = 2.06), respectively (Figure 4).

For MAP, the observed reductions were 3% (*p* = 0.999, *g* = 0.22), 15% (*p* = 0.2109, *g* = 1.46), 28% (*p* = 0.0016, *g* = 2.26), 30% (*p* = 0.0002, *g* = 2.79), 35% (*p* < 0.0001, *g* = 4.68) and 38% (*p* < 0.0001, *g* = 2.23) at the same doses. However, heart rate was not significantly affected (Figure 5).

Hedges’ *g* values estimate the magnitude of observed effects, with higher values indicating larger effect sizes. A *g* value between 0.2 and 0.3 is typically considered a small effect, ∼0.5 a medium effect, and >0.8 a large effect. Based on the calculated Hedges’ *g* values, the results suggest that AERu exerts a significant hypotensive effect. This effect is particularly pronounced at doses of 10, 20, 30 and 40 mg/kg body mass, where reductions in blood pressure were both statistically significant and associated with large effect sizes.

### 3.4 Vasodilator effect of the AERu

**FIGURE 6:**
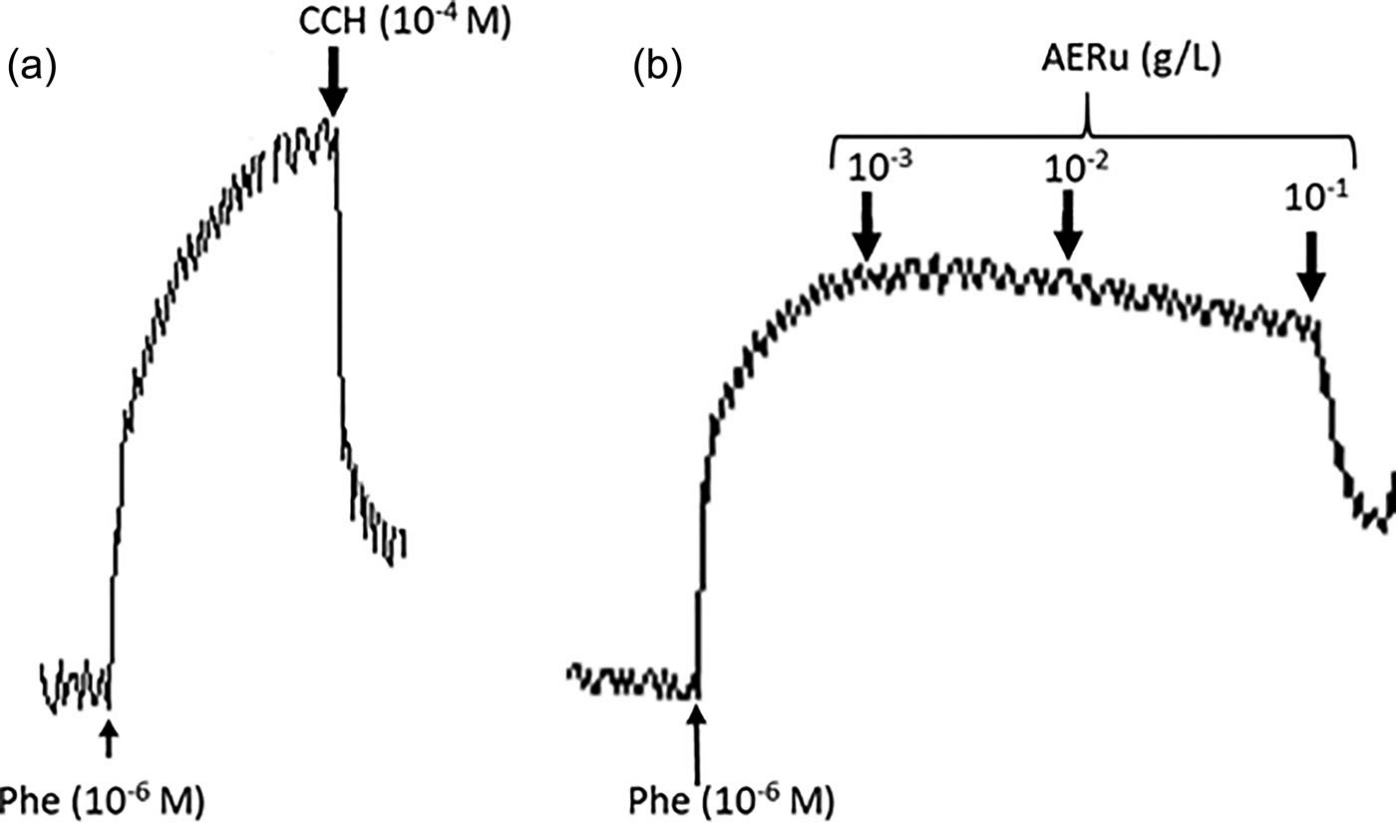
Original traces of the vascular effect of carbachol (CCH; 10^−4^ M) and cumulative doses (10^−3^, 10^−2^ and 10^−1^ g/L) of the aqueous extract of blackberry leaves (AERu). (a) Carbachol induces a 75% relaxation in functional vascular endothelium. (b) Vasorelaxant effect of the AERu at a dose of 10^−1^ g/L.

**FIGURE 7:**
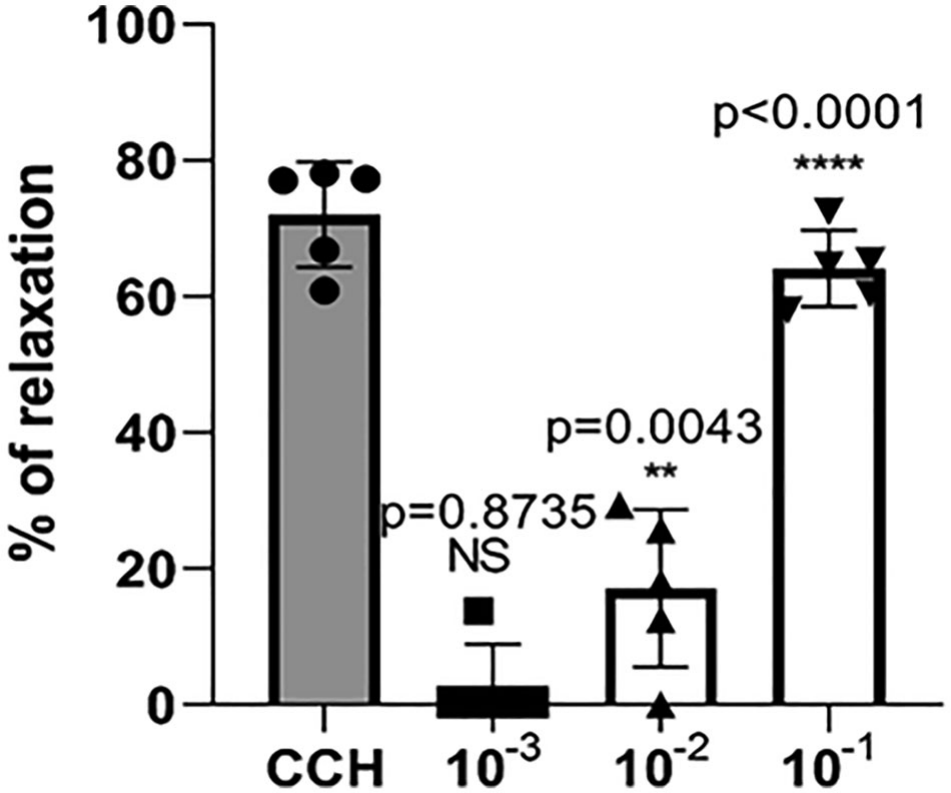
The vasorelaxant effect of cumulative doses of aqueous extract of blackberry leaves (AERu) on phenylephrine‐precontracted aorta. Data are expressed as mean ± SD (*n* = 5 rats per group). ^**^ *p* < 0.01, ^****^ *p* < 0.0001 versus phenylephrine. Abbreviations: CCH, carbachol; NS, not significant.

AERu induced a dose‐dependent vasorelaxant effect. Cumulative doses of this extract (10^−3^, 10^−2^ and 10^−1^ g/L) resulted in relaxations of 2.75%, 17.11% and 64.13%, respectively, in PHE‐precontracted aortic rings. These effects were initially tested on a functional vascular endothelium and compared with those of carbachol (Figures 6 and 7).

## 4 DISCUSSION

Mugwort and blackberry are recognized for their nutritional and medicinal properties; however, their impact on gene expression has never been analysed. We performed a functional analysis for mugwort and blackberry by carrying out a nutrigenomic analysis on mice that had ingested a solution prepared from the leaves of blackberry or the aerial part of mugwort. We observed that the effects of mugwort were reflected in the deregulation of 52 genes that are involved in cell division and cell proliferation, cell adhesion, cell differentiation, apoptosis, immunity, cardiovascular functions, digestion, intracellular transport, metabolism, nervous system function and development, and signalling and regulation. Blackberry elicited similar responses but more pronounced than mugwort, because 77 genes involved in the same functions were found to be deregulated (Figures 2 and 3).

Initially, we reviewed various studies on these genes to understand their involvement in the physiology of the organism (Supplementary file). Then we selected the genes whose deregulation observed with mugwort or blackberry treatment suggested: (1) a vasodilator and hypotensive effect; (2) an antitumoral effect; (3) an antimicrobial and anti‐inflammatory effect; and (4) an impact on eating behaviour.

### 4.1 The vasodilator and hypotensive effect

Vessel lumen expansion is a complex morphogenetic event that relies not only on cell proliferation and differentiation but also on cell–cell junctional communication that is fundamental to vessel formation or morphological changes. Mugwort has already been studied for its antihypertensive effects (Dib & El Alaoui‐Faris, 2019), and blackberry was already proposed by the ethnobotanical studies of Morocco for different cardiovascular treatments, based on its clinical effects (Fakchich & Elachouri, 2021). In our study, we found that mugwort and blackberry treatments stimulated the upregulation of 6 and 12 genes, respectively, involved in cell division and proliferation; 1 and 3 genes, respectively, involved in cell differentiation; and 5 and 6 genes, respectively, involved in cell adhesion (Figure 2). From these functional groups, we selected several genes that were described to be involved mainly in vessel lumen expansion that suggest a vasodilatory and hypotensive effect. *Amotl2* (Angiomotin‐like 2; Figure 2, no. 9) found to be more upregulated by the blackberry, has been described as being involved in endothelial cell functions for the coordination of cellular morphogenesis consistent with aortic lumen expansion and function (Hultin et al., 2014). It was shown that amotL2/VE‐cadherin junctional complex is part of the machinery translating junctional signals to actin cytoskeleton‐driven changes in cell shape, which are essential for aortic lumen expansion. As additional support for this hypothesis, we highlighted cadherin coding gene (*Cdh1*) and other similar genes involved in cell–cell adhesion and communication as upregulated by both treatments (Figure 2, nos 16–21). Likewise, we found *Tfcp2l1* from the group of genes involved in cell differentiation (Figure 2, no. 14). This gene encodes a transcription factor involved in epithelial cell maturation, suggesting a potential vasorelaxant effect. We found it to be more upregulated by the blackberry treatment (FC ranging from 1.55 to 2.06) than by the mugwort treatment (FC ranging from 1.22 to 1.54). We found that after a plant diet, several genes involved in cardiac and circulatory system morphology and activity were deregulated (Figure 2, nos 22–25). This is the case for *Dsp*, which codes for desmoplakin. This protein is involved in cell adhesion but also in ventricular compact myocardium morphogenesis and cardiac performance (Stevens et al., 2022). *Ahnak* codes for a protein of the cardiovascular system that enables structural molecule activity conferring elasticity. This gene was described as being involved in vascular remodelling by re‐endothelialization and thus playing a protective role in vascular healing (Haase et al., 2017). *Prcp* codes for angiotensinase C, which is involved in blood pressure reduction (Maier et al., 2017). *Dsp*, *Ahnak* and *Prcp* seem to be upregulated similarly by both tested plants, suggesting that the cardiac muscle and the circulatory system would become more elastic and the blood pressure further reduced. *Smtnl2* promotes epithelial morphogenesis by stabilizing actin filaments (Hachimi et al., 2021) and binds tropomyosin, which is involved in vasoconstriction and contributes to hypertension (Xu et al., 2021). In our case, both mugwort and blackberry downregulated *Smtnl2*, suggesting a potential hypotensive effect.

Through *ex vivo* experiments, we demonstrated that AERu induces a dose‐dependent hypotensive effect. The results obtained (Figures 4 and 5) showed that, starting from a dose of 20 mg/kg of body mass, this effect became significant for all measured parameters (SBP, DBP and MAP) in normotensive rats, while heart rate remained unchanged. Using an identical approach for mugwort, a previous study (Dib, Tits et al., 2017) found the hypotensive effect to be significant from a dose of 5 mg/kg of body mass across all measured parameters. Similar to blackberry, heart rate was not affected. This suggests that the hypotensive effect of these two plants operates through the vascular pathway, while the cardiac pathway is excluded from this effect, and seems twice as pronounced for mugwort as for blackberry.

Furthermore, a vasorelaxant effect of AERu on intact aortic rings was demonstrated (Figures 6 and 7). The obtained results indicated a highly significant vasodilatory effect, reaching a maximum relaxation of 64.13% at a dose of 10^−1 ^g/L. This effect is comparable to that of carbachol, an analogue of acetylcholine, causing dilatation of blood vessels through the release of a vasorelaxant agent, nitric oxide (NO), by the endothelium. Likewise, a vasodilatory effect of the aqueous extract of mugwort was observed (Dib, Tits et al., 2017) for multiple doses (10^−2^, 10^−1^, 1 and 2 g/L), with a maximum relaxation of 95% noted at the dose of 2 g/L. The vasorelaxant effect was much more pronounced for blackberry, because the dose required for maximal vasodilatory effect was 20 times lower than that needed for mugwort.

We observed that the hypotensive and vasorelaxant effects varied in intensity between the two plants. The more pronounced vasorelaxant effect of blackberry could be attributed to the increased upregulation of *Tfcp2l1*, which is involved in epithelial cell maturation, and might result from a different phytochemical composition.

We compared our phytochemical analysis of AERu with previous studies on mugwort (Dib, Angenot et al., 2017; Dib, Tits et al., 2017) to target better the molecules with a hypertensive role. Based on knowledge about the role of polyphenols (Dall'Acqua et al., 2008; Flamini et al., 2002; Martins et al., 2014; Panizzi et al., 2002), we focused on chlorogenic acids, tannins and flavonoids, which have established hypertensive effects. Concerning phenolic acids, different forms of caffeoylquinic acids (5‐caffeoylquinic acid and 3‐caffeoylquinic acid) and coumaroylquinic acids (3‐*p*‐coumaroylquinic acid and 3‐*p*‐coumaroylquinic acid derivative) were found in AERu (Figure 1; Table 2). Caffeoylquinic acids (3,5‐dicaféoylquinic acid, 4,5‐dicaféoylquinic acid and 3,4‐dicaféoylquinic acid) and coumaroylquinic acids were also identified in the mugwort (Dib, Angenot et al., 2017; Dib, Tits et al., 2017). These compounds, along with their metabolites, including ferulic acid and caffeic acid, have already been described for their antihypertensive effects (Zhao et al., 2012). In vitro, ferulic acid has been shown to improve vasodilatation in rat aortic rings by increasing the bioavailability of NO induced by acetylcholine (Suzuki et al., 2007). Moreover, in spontaneously hypertensive rats, oral administration of a dose of 9.5 mg/kg body weight of ferulic acid decreased SBP by 30 mmHg compared with the initial value (Ardiansyah et al., 2008). Generally, chlorogenic acids are known to reduce oxidative stress in blood vessels by decreasing the production of superoxide anions through the enzyme NADPH oxidase (Zhao et al., 2012). Oxidative stress contributes to hypertension owing to the reaction of these superoxide anions with NO, thereby reducing its bioavailability. It also contributes to an increased expression of cyclooxygenases, promoting the formation of vasoconstrictive agents (Stoclet & Schini‐Kerth, 2011). Based on these studies, we can identify caffeoylquinic acids and coumaroylquinic acids as key contributors to the effects of both blackberry and mugwort.

We identified a significant amount of tannins and flavonoids in AERu (Figures 1 and 2; Tables 1 and 2) and compared its composition with previous analyses of mugwort (Dib & El Alaoui‐Faris, 2019; Dib, Tits et al., 2017) (Table 3). In AERu, rubanthrone A (an anthrone) and a galloyl‐*bis*‐HHDP glucose derivative (a tannin) were detected. Among the flavonoids, afzelin, quercetin‐3‐*O*‐β‐d‐glucuronide and kaempferol derivatives were identified. In mugwort, cinnamtannin B1 was identified as a tannin, while vicenin 2, quercetin‐3‐*O*‐β‐d‐glucuronide and kaempferol derivatives were detected as flavonoids.

**TABLE 3:** Comparative composition of tannins and flavonoids identified in blackberry and mugwort.

Class of molecules	Molecules identified in blackberry (this study)	Molecules identified in mugwort (Dib & El Alaoui‐Faris, 2019; Dib, Tits et al., 2017)
Tannins	Galloyl‐*bis*‐HHDP glucose derivative	Cinnamtannin B1
Flavonoids	Afzelin	Vicenin 2
	Quercetin‐3‐*O*‐β‐d‐glucuronide	Quercetin‐3‐*O*‐β‐d‐glucuronide
	Kaempferol derivatives	Kaempferol derivatives

Concerning tannins, 1,2,3,6‐tetra‐*O*‐galloyl‐β‐d‐glucose has been described as reducing blood pressure by inhibiting the angiotensin‐converting enzyme, which transforms angiotensin I into angiotensin II, a vasoconstrictive agent (Liu et al., 2003). The galloyl‐*bis*‐HHDP glucose derivative found in AERu is a type of ellagitannin, as is 1,2,3,6‐tetra‐*O*‐galloyl‐β‐d‐glucose, which could be related to similar effects. Ellagitannins were not found in *Artemisia* species; however, another tannin, cinnamtannin B1, was studied in mugwort, contributing mostly to the antioxidant properties of the plant (López et al., 2008).

Regarding flavonoids, their beneficial effects on cardiovascular diseases have attracted renewed interest in recent years (Ma et al., 2018; Shang et al., 2018). A study demonstrated that the consumption of fruits and vegetables rich in flavonoids improved blood vessel function in men at risk of cardiovascular diseases by increasing plasma nitric oxide levels and reducing inflammation (Macready et al., 2014). At the vascular level, In vitro, quercetin reduced the contractile response of isolated smooth muscle cells from rats induced by elevated levels of calcium, potassium and phorbol myristate acetate (Duarte et al., 1993). Other studies have shown that quercetin improved endothelial function In vitro by inducing endothelium‐dependent vasodilatation mediated by endothelium‐derived hyperpolarizing factor and NO (Benito et al., 2002; Khoo et al., 2010). An *ex vivo* study demonstrated that epigallocatechin gallate administered in the isolated mesenteric vascular bed of rats caused a dose‐dependent relaxation. Thus, it stimulated NO production by endothelial cells isolated from bovine aortas (Kim et al., 2007). A previous study reported that quercetin 3‐*O*‐β‐d‐glucuronide could prevent arteriosclerosis and could be used to control hypertension (Ohara et al., 2013). Cechinel‐Zanchett et al. (2020) also demonstrated the vasorelaxant effects of the flavonoid‐rich fraction of *Bauhinia forficata* in normotensive and hypertensive aortic rings. This activity was attributed to kaempferitrin, the biotransformation of which produced the bioactive compound afzelin. Furthermore, the diuretic and renal protective effect of afzelin in normotensive and hypertensive rats was recently reported (Cechinel‐Zanchett et al., 2020).

Based on these arguments, we can nominate quercetin‐3‐*O*‐β‐d‐glucuronide, kaempferol‐3‐*O*‐β‐d‐glucuronide, kaempferol‐3‐*O*‐α‐l‐rhamnopyranoside and other kaempferol derivatives found in blackberry and mugwort extracts as significant contributors to the antihypertensive effects of both plants. The exclusive presence of galloyl‐*bis*‐HHDP glucose derivatives in blackberry could explain its enhanced hypotensive properties. In addition to the qualitative comparison, a precise quantitative assessment of phytochemicals in both plants could offer further insights and remains a key direction for our research.

### 4.2 Antiproliferative effect

An anti‐tumour effect was suggested by the deregulation of several genes involved in cell division and cell adhesion regulation, after the ingestion of a mugwort and/or blackberry diet supplementation. These genes have been described as involved at the same time in tumour suppression or in the prevention of tumour proliferation.

Two genes in the group of upregulated genes involved in cell division caught our attention: *Amotl2* (Figure 2, no. 9) and *L3mbtl1* (Figure 2, no. 5). They are both more upregulated in the blackberry treatment. *Amotl2* was described with a dual tumour suppressive function by targeting both Yap (known as anti‐apoptosis transcription factor) and Akt (which contributes to impaired apoptosis in cancer) (Han et al., 2017). L3mbtl1 polycomb protein was described as a candidate tumour suppressor in myeloid disorders, and its expression was described as essential for genome stability (Gurvich et al., 2010).

We also observed the deregulation of several genes known to be involved in cell adhesion, which is related to cancer metastasis. The loss of cell–cell adhesion in metastatic tumour cells allows them to escape their site of origin and spread through the circulatory system (Okegawa et al., 2004). Of these genes, we found *Sh3kbp1*, *Frmd5* and *Adrm1* (Figure 2, nos 16–18) to be upregulated in both treatments, and they are described in the literature to be involved in cancer prevention (Keller et al., 2018; Pourhaghighi et al., 2020; Simins et al., 1999). We can hypothesize that mugwort and blackberry might trigger an antitumoural effect, because mugwort can influence at least three genes and blackberry at least five genes that have already been described as involved in this response.

Mugwort has already been studied for its antioxidant effect (Dib & El Alaoui‐Faris, 2019), as have blackberry fruits, which contain high levels of phenols, flavonols and anthocyanins and are well‐reputed scavengers and inhibitors of free radicals (Jiao & Wang, 2000). Blackberry leaves are also known to exhibit chemopreventive effects in rats (Stoner et al., 2006), antioxidant (Buricova et al., 2011) and antiproliferative capacities (Triggiani et al., 2012). Our observations on nutrigenomic responses are fully consistent with these benefits in mice, and the antiproliferative effect was clearly suggested by the gene expression profiles.

### 4.3 Antimicrobial and anti‐inflammatory effects

Eight genes involved in immune responses were upregulated by both plant treatments (Figure 2, nos 29–36). *Cd300lf*, *Sigirr* and *Mirt2* were described as being involved in inhibition or negative regulation of inflammatory response (Du et al., 2017; Giannoudaki et al., 2021; Keswani et al., 2020), and their upregulation suggests an anti‐inflammatory effect of both plants. The other genes from this group, *Fcgr2b*, *Cst11*, *Trav3‐3*, *Ppnr* and *Pglyrp3*, are known to be involved in responses to bacteria (Anania et al., 2019; Diez‐Roux et al., 2011; Fan et al., 2014; Ghadimi et al., 2021). Their upregulation suggests that mugwort and blackberry could increase the defence against bacteria.

Many clinical anti‐inflammatory and antibacterial effects have already been registered for mugwort (Dib & El Alaoui‐Faris, 2019) and for blackberry (Abachi et al., 2013; Meziti et al., 2018; Riaz et al., 2011; Yang et al., 2014). Our work demonstrates that these clinical effects can now be linked to nutrigenomic responses in gene expression profiles.

### 4.4 Eating behaviour

Nutrigenomic analyses showed that nine genes involved in nervous system development and functions (Figure 2, nos 37–45) and six genes involved in digestive functions (Figure 2, nos 46–51), were upregulated mostly by the blackberry diet. Disabled‐1 (*Dab1*) encodes a key regulator involved in neurogenesis. DAB1 functions in a signalling pathway that controls cell positioning in the developing brain and is required for normal cognitive function (Howell et al., 1997). *Dab1* expression was upregulated by both the tested plants. The remaining genes in this group (*Tas2r123*, *Olfr142*, *Olfr808*, *Olfr92*, *Olfr825*, *Obp2b*, *Vmn1r63* and *Vmn2r109*) code for taste, olfactory or vomeronasal receptors and are upregulated mostly by the blackberry diet. It is known that the taste, olfaction and pheromonal activation of the vomeronasal organ elicits physiological and behavioural changes in mammals (Pantages & Dulac, 2000). Our results suggest that mugwort and especially blackberry could influence palatability and potentially alter appetite and food intake‐related behaviour.

We attempted to establish a connection with the DEGs involved in digestion and noticed that two genes related to glucose metabolism and homeostasis were upregulated: *Gcg* and *Mbd5*. *Gcg* encodes a protein that is processed to generate glucagon and two other glucagon‐like peptides, GLP1 and GLP2. Glucagon is involved in glucose metabolism by stimulating gluconeogenesis and glycogenolysis, and GLP1 is known as the hormone incretin, with an insulinotropic action and feeding inhibition (Wewer Albrechtsen et al., 2023). The second gene, *Mbd5*, was described as an essential factor for maintenance of glucose homeostasis (Du et al., 2012). Several genes encoding transporters appear to be stimulated particularly by the blackberry diet. Among these are *Slc5a1* and *Slc2a4*, which code for glucose transporters involved in glucose homeostasis processes.

These nutrigenomic results could be associated with many clinical observations that show an antidiabetic effect of mugwort (Dib & El Alaoui‐Faris, 2019) and ‐ effective blood glucose lowering by blackberry leaves (Jouad et al., 2002; Ştefănuţ et al., 2013; Zia‐Ul‐Haq et al., 2014). Our study indicates that the effect of these plants occurs via modifications in gene expression that impact eating behaviour and glucose metabolism.

Despite the progress made with these analyses, several limitations must be acknowledged. First, the findings are based on animal models, and further validation in human clinical trials is necessary to confirm the translatability of these results. Second, the specific molecular mechanisms underlying the gene deregulation and the interaction between plant metabolites and target pathways remain to be elucidated. Additionally, the potential variability in plant metabolite content owing to environmental factors, cultivation conditions and plant part usage was not addressed in this study. Finally, the sample size for certain analyses might limit the generalizability of the conclusions. All the same, our observations lay a solid foundation for further research aimed at developing functional foods and plant‐based nutraceuticals specifically designed to promote cardiovascular health.

## 5 CONCLUSION

This work highlights that both blackberry and mugwort exhibit vasorelaxant and hypotensive effects. These effects were related to the deregulation of genes such as *Amotl2*, *Cdh1* and *Tfcp2l1*, which are involved in vessel lumen expansion, in addition to *Dsp*, *Ahnak*, *Prcp* and *Smtnl2*, which are involved in circulatory system morphology and function. The deregulation of these genes might be attributed to plant metabolites such as caffeoylquinic and coumaroylquinic acids, quercetin and kaempferol derivatives found in both plants. A significant difference was observed in the vasorelaxant effect, which was 20 times greater during blackberry treatment compared with mugwort. The exclusive presence of the galloyl‐*bis*‐HHDP glucose derivative in AERu, known for its hypotensive properties, along with the more pronounced upregulation of *Tfcp2l1* (implicated in epithelial cell maturation) during blackberry treatment in mice, could explain the enhanced cardiovascular effects of blackberry compared with mugwort.

This study demonstrates that blackberry and mugwort, used as medicinal plants, are valuable sources of active compounds with cardiovascular effects. Our findings support the traditional use of both plants for treating hypertension and suggest their potential applications in cancer prevention, inflammation reduction and appetite regulation.

## AUTHOR CONTRIBUTIONS

Afaf Mehiou: investigation, formal analysis, data curation, resources, writing—review & editing. Anca Lucau‐Danila: conceptualization, investigation, formal analysis, data curation, methodology, writing—original draft preparation. Zachee L. E. Akissi: investigation, formal analysis, data curation, methodology, writing—original draft preparation. Chaimae Alla: investigation, formal analysis, data curation, resources, writing—review & editing. Nourelhouda Bouanani: resources, writing—review & editing. Abdelkhaleq Legssyer: resources, writing—review & editing. Jean‐Louis Hilbert: conceptualization, supervision, writing—review & editing. Sevser Sahpaz: conceptualization, supervision, project administration, writing—review & editing. Abderrahim Ziyyat: conceptualization, supervision, funding acquisition, project administration, writing—review & editing. All authors read and approved the final version of the manuscript and agree to be accountable for all aspects of the work in ensuring that questions related to the accuracy or integrity of any part of the work are appropriately investigated and resolved. All persons designated as authors qualify for authorship, and all those who qualify for authorship are listed.

## CONFLICT OF INTEREST

The authors declare that there is no conflict of interest regarding the publication of this article.

## Supporting information




**Supplementary file**. Description and function of deregulated genes observed by nutrigenomic studies after AEAc and AERu ingestion.

## Data Availability

Microarray data have been submitted to the NCBI GEO: archive for functional genomics data with the accession number GSE251733.
